# Effect of different spatial normalization approaches on tractography and structural brain networks

**DOI:** 10.1162/netn_a_00035

**Published:** 2018-09-01

**Authors:** Clint Greene, Matt Cieslak, Scott T. Grafton

**Affiliations:** Signal Compression Lab, Department of Electrical and Computer Engineering, University of California, Santa Barbara, CA, USA; Action Lab, Department of Psychological and Brain Sciences, University of California, Santa Barbara, CA, USA; Action Lab, Department of Psychological and Brain Sciences, University of California, Santa Barbara, CA, USA

**Keywords:** Diffusion MRI, Tractography, Spatial normalization, Brain networks, Connectomes

## Abstract

To facilitate the comparison of white matter morphologic connectivity across target populations, it is invaluable to map the data to a standardized neuroanatomical space. Here, we evaluated direct streamline normalization (DSN), where the warping was applied directly to the streamlines, with two publically available approaches that spatially normalize the diffusion data and then reconstruct the streamlines. Prior work has shown that streamlines generated after normalization from reoriented diffusion data do not reliably match the streamlines generated in native space. To test the impact of these different normalization methods on quantitative tractography measures, we compared the reproducibility of the resulting normalized connectivity matrices and network metrics with those originally obtained in native space. The two methods that reconstruct streamlines after normalization led to significant differences in network metrics with large to huge standardized effect sizes, reflecting a dramatic alteration of the same subject’s native connectivity. In contrast, after normalizing with DSN we found no significant difference in network metrics compared with native space with only very small-to-small standardized effect sizes. DSN readily outperformed the other methods at preserving native space connectivity and introduced novel opportunities to define connectome networks without relying on gray matter parcellations.

## Author Summary

Direct streamline normalization (DSN) directly warps the streamlines into any template space by using the transformations output from Advanced Normalization Tools (ANTs). DSN overcomes the limitations of diffusion weighted images (DWI) spatial normalization. It allows DWIs to be acquired with any desired sampling scheme. Fiber orientation distributions (FODs) or orientation distribution functions (ODFs) can also be reconstructed using any desired method and streamlines generated using any algorithm. Most importantly, it avoids the problem of generating tracts from FODs or ODFs that have become distorted because of spatial normalization. Our results show that DSN has minimal influence on tractography measures such as tract count and structure and does not significantly alter structural networks with only very small to small effect sizes. We have developed a framework in Python that works with most diffusion software platforms. It is available at http://github.com/clintg6/DSN.

## INTRODUCTION

Over the past two decades, diffusion-weighted magnetic resonance imaging (DW-MRI) techniques have been used to noninvasively explore fiber bundle architectures in the brain by leveraging local estimates of anisotropy to reconstruct streamlines (virtual proxies of sets of collinear fibers tracts). These techniques have become an essential toolset for the diagnosis of developmental brain disorders (Chang & Zhu, 2013), preoperative planning in neurosurgery (Golby et al., 2011; Jenabi et al., 2014), and the study of brain connectivity in healthy individuals (Van Essen et al., 2012). To enhance population-based analysis of white matter morphology, it is desirable to spatially normalize the reconstructed “fiber tracts” (herein called streamlines) into a standardized neuroanatomical space. Spatial normalization is routinely used in voxel-based morphometry, structural MRI, and resting-state fMRI. Embedding streamline connections into these morphologic and functional databases would be invaluable for furthering our understanding of structure-function relationships of the brain (de Schotten et al., 2016). Spatially normalized tractography is important because it enables the characterization of differences in white matter morphology due to development, genetics, disease, or injury across populations. For example, a method known as local termination pattern analysis (LTPA) leverages normalized tractography datasets to compare white matter morphology across populations by itemizing the pairwise cortical region termination connectivity for the subset of streamlines passing through a small cluster of voxels (Cieslak & Grafton, 2014; Cieslak et al., 2015). The termination patterns can be used to distinguish among groups. Spatially normalized tractography has also been used to construct structural connectivity networks in a standardized way (Jarbo & Verstynen, 2015; Gu et al., 2015; Molesworth et al., 2015; Muraskin et al., 2016; Donos et al., 2016). Such methods also necessitate accurately normalized streamlines. Once normalized, there is opportunity to cluster fascicles across populations and to generate new types of cortical parcellations driven by white matter trajectories.

Spatial normalization of diffusion data typically begins by registering the set of diffusion weighted images (DWIs) to a higher resolution T1 weighted anatomic scan with a rigid body transformation. The latter is then registered to a template atlas, such as the Montreal Neurological Institute (MNI) atlas by using one of a variety of algorithms that typically utilize both linear and higher order nonlinear transformations with many degrees of freedom. Extensive prior work has compared the accuracy of different nonlinear deformation algorithms (Klein et al., 2009) for mapping T1 weighted images, with leading performers including SyN, ART, IRTK, and DARTEL. These are notably superior to the dated nonlinear deformation algorithms used in SPM and FSL. These algorithms are typically optimized over a similarity metric such as mean squared difference, cross-correlation, or mutual information. In this paper, we employed SyN. Once the mapping between the diffusion scans and template space has been determined, there are two basic approaches to create spatially normalized streamlines. The first is to transform the underlying diffusion information into the atlas and then perform streamline reconstruction. The second is to create the streamlines with respect to the original diffusion scans and then warp these streamlines to the atlas space. We first review the advantages and disadvantages of the former approaches (warping then streamline construction) and then propose the second approach (streamline construction followed by streamline warping) as a major improvement.

One approach for generating streamlines after normalization of diffusion information into template space involves reorientation of either diffusion tensors from diffusion tensor imaging (DTI) scans or fiber orientation distributions (FODs) derived from high angular resolution diffusion imaging (HARDI) scans. The tensors or FODs rather than diffusion scans are subsequently reoriented (Alexander et al., 2001; Zhang et al., 2006; Hong et al., 2009; Raffelt et al., 2011, 2012). Irrespective of which of these sampling schemes are used, spatial reorientation at this step has undesirable effects. For example, prior work investigated the adverse effects of nonlinearly warping DTI data by evaluating the consistency of the geometric shape of specific white matter pathways such as the corpus callosum and cingulum bundle (Adluru et al., 2016). Whole brain tractography in both the subject’s native space and after tensor reorientation in the template space were performed and regions of interest (ROIs) were used to extract known pathways. To measure the impact of spatial normalization on the shape of the tracts, the authors compared the overlap of the rasterized masks of the pathways. Unsurprisingly, they found the least amount of overlap between native and normalized pathways near the gray-white boundary, where white matter pathways begin to branch.

For FOD reorientation, another undesirable effect is that the maxima in the native FODs no longer corresponds to the maxima in the reoriented FODs (Christiaens et al., 2012). This is because the reorientation introduces dimple artifacts hypothesized to be from the negative lobes in the Gibbs ringing. This directly affects subsequent deterministic and probabilistic fiber tractography performed in the template space, producing streamlines that do not match the original streamlines from native space. Moreover, streamline distributions generated from probabilistic tractography performed on reoriented FODs are deflected with respect to the native tract distribution (Christiaens et al., 2012). Although the latest work in FOD reorientation (FODR) using apodized point spread functions (PSFs) overcomes the dimple artifacts, slight distortions are still introduced into the spread of the fiber populations (Raffelt et al., 2012). On average the angular error between peak orientations is ∼8° (Raffelt et al., 2012). The error in peak orientation accumulates into large errors when performing tractography after normalization (Colon-Perez et al., 2015). Moreover, an obvious drawback of these types of solutions is that they depend specifically on the diffusion sampling and reconstruction method used and do not generalize easily to other diffusion methods. Although FODR software was recently made public, it has not been applied to the study of population-based tractography or structural network analysis.

Another approach for generating streamlines after normalization is to apply the nonlinear spatial transformations to each of the DWIs and locally reorient the b-vector using the Jacobian of the deformation field to reconstruct the orientation distribution function (ODF). This approach has the merit of being generally applicable to any type of diffusion weighted scan, irrespective of the sampling scheme. Diffusion spectrum imaging (DSI) Studio (http://dsi-studio.labsolver.org) has a publicly available implementation of this approach known a q-space diffeomorphic reconstruction (QSDR) that works with HARDI and DSI sampling schemes (Yeh & Tseng, 2011). A multitude of work has used QSDR for comparing per-subject tractography and for constructing normalized structural networks (Cieslak & Grafton, 2014; Cieslak et al., 2015; Jarbo & Verstynen, 2015; Gu et al., 2015; Molesworth et al., 2015; Muraskin et al., 2016; Donos et al., 2016). However, the QSDR algorithm suffers from some limitations. First, it relies on the dated SPM2 spatial normalization algorithm that has been shown to have inferior registration performance compared with newer methods (Klein et al., 2009). Secondly, it relies on a single contrast modality, the quantitative anisotropy (QA) volume estimated from generalized q-sampling imaging (GQI) reconstructed native space diffusion data (Yeh & Tseng, 2011), to register the native diffusion data to a QA template derived from diffusion scans with similarly low contrast and significant spatial inhomogeneity in the occipital lobe. Third, the mean angular error on simulated vertical fibers is 2.27° and likely higher with subject data. Critically, the nonlinear transformations required to transform brains of various shapes and sizes into a standardized space invariably introduces noise into the QSDR ODFs (Powell et al., 2018). Consequently, the resulting tractograms suffer from similar types of distortions that occur when tracking through reoriented tensors or FODs.

Given the problems that arise from spatially normalizing diffusion information prior to streamline construction, we sought to determine if direct streamline normalization (DSN), where the streamlines are created first, in the same space as the diffusion scans, followed by the warping of these streamlines into the template space would yield more precise results. In this case, the deformation fields from the normalization of each subject to the template are used for warping the streamlines (Hua et al., 2008; Thottakara et al., 2006). DSN confers multiple potential advantages. DWIs can be acquired with any desired sampling scheme. Diffusion tensors, FODs, or ODFs can also be reconstructed using any desired method and streamlines generated using any algorithm. Most importantly, it avoids the problem of generating streamlines from reoriented diffusion tensors, FODs, or ODFs that are distorted relative to their native counterparts, which substantially reduces errors in tract morphology relative to the subject’s native structure.

In this paper, we assessed the precision of the two publically available DWI spatial normalization techniques (FODR and QSDR) and direct streamline normalization for warping tractography data to a standardized atlas. We investigated the impact of the different normalization schemes on structural brain networks and topologic properties from a subject pool of 417 Human Connectome Project (HCP) subjects. For DSN, we utilized a publically available symmetric diffeomorphic algorithm symmetric groupwise normalization (SyGN) using Advanced Normalization Tools (ANTs), known for its registration accuracy and performance (Klein et al., 2009), to construct custom high-resolution multimodal templates and to directly normalize the streamlines (Avants et al., 2010). In our approach, we used T1w, T2w, and generalized fractional anisotropy (GFA) images to enhance the fidelity and contrast for the template generation and normalization for improved cortical and white matter alignment. Our comparison is distinct from prior efforts in this area (Adluru et al., 2016) because we used HARDI data that can resolve multiple fiber crossings, we analyzed at a finer level than pathways and take into account how branching impacts structural networks.

We relied on several figures of merit to compare precision of the streamline normalization methods. First, we visually compared the quality of known white matter tracts such as the corticospinal tract before and after normalization. Second, we applied a set of gray matter regions of interest from native anatomic space to each subject’s streamline data, also in native space. From this, native space connectivity matrices were extracted and used to estimate native space network properties. If the same gray matter regions were normalized and reapplied to the normalized streamlines, then all of the network properties measured in native space should be preserved in the atlas space. Similarly, the connectivity matrices themselves should be similar. In addition to quantifying dissimilarity statistically, we also reported standardized effects sizes to capture the magnitude of error induced by each normalization method. We found large to very large effect sizes in the same subject’s network metrics after normalization with QSDR and FODR. In contrast, DSN has only very small-to-small effects on the network metrics. Third, we capitalized on the availability of twin data within the HCP database to further assess the precision of the different methods. We used pairs of identical twins, fraternal twins, nontwin siblings, and nonrelated subjects to characterize the inherent variability in structural brain networks. Identical twins have connectivity matrices that are significantly more similar to each other than strangers are to each other. This pattern of similarity should also be observed after successful spatial normalization. Here too, we showed that preserving genetic influences on network metrics can be heavily influenced by the spatial normalization approach. The effects can be dramatic, with some methods changing the same subject’s connectivity and network metrics after normalization to a relative distance that is comparable to a nontwin family member rather than to themselves. These comparisons demonstrate the significant gains that directly normalizing the streamlines achieves compared with the other methods at preserving the native tract structure and properties of structural brain networks. Instructions for downloading DSN software that provides a universal framework that works with most diffusion software platforms, algorithms, and that makes use of state-of-the-art spatial normalization techniques for directly normalizing the streamlines are provided.

## MATERIALS AND METHODS

### Preprocessing

#### Imaging data.

The dataset was collected as part of the Washington University–University of Minnesota Consortium Human Connectome Project (Van Essen et al., 2013). The data used was from the S500 release, consisting of structural and diffusion data from 489 participants. Data from 49 subjects were not used because the number of diffusion volumes was incomplete or suffered from artifacts. The structural and diffusion data were collected on 3T Connectome Skyra system (Siemens, Erlangen, Germany) over a specified set of spatial and angular resolutions. The diffusion volumes were collected with a spatial resolution 1.25 × 1.25 × 1.25 mm, using three shells at b = 1,000, 2,000, and 3,000 s/mm^2^ with 90 diffusion directions per shell and 10 additional b0s per shell. The diffusion data was corrected for geometric and eddy current distortions, using information from acquisitions in opposite phase-encoding directions, as well as head motion (Glasser et al., 2013). The high-resolution structural T1w and T2w volumes were acquired on the same scanner at 0.7-mm isotropic resolution (Glasser et al., 2013).

#### Multimodal template construction.

**Figure 1.**
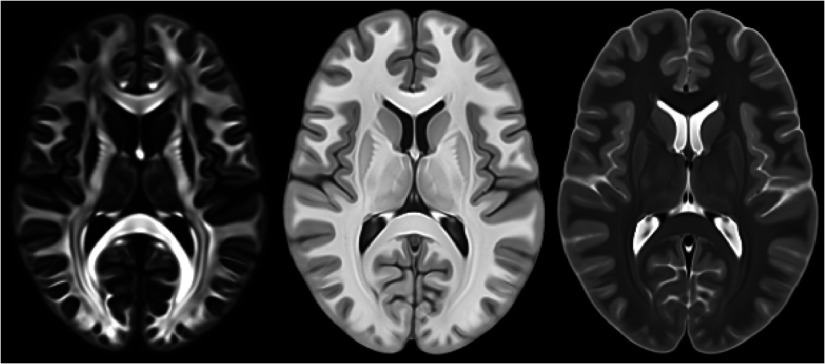
High-resolution multimodal templates generated using SyGN from 40 HCP subjects. From left to right: GFA, T1w, and T2w. SyGN leverages information from the multiple modalities to create the most optimal and unbiased template with respect to shape and appearance. Notice that SyGN preserves the shared sharp features across the subjects that are visible in the caudate, putamen, thalamic, and frontal regions while optimally representing the finest shape differences in the occipital area.

GFA volumes for each subject were extracted from their GQI reconstructed HARDI data in DSI Studio. Previously skull-stripped, aligned, and distortion-corrected T1w and T2w volumes were obtained for each subject (Glasser et al., 2013) and then rigidly registered to the subject’s GFA volume. ANTs symmetric groupwise normalization (SyGN) method was used to construct a custom multimodal population-specific brain template from 40 HCP subjects by using five iterations (Avants et al., 2010). The subjects were chosen through stratified random sampling to give each racial, gender, and handedness group a representation in the template. It has been previously shown that normalization to a custom template improves localization accuracy, reduces bias in statistical testing, and ultimately yields more biologically plausible results during analysis as opposed to using a standardized MNI template that was not constructed from the subject pool (Kim et al., 2008). Each subject’s image set input into SyGN consisted of GFA, T1w, and T2w volumes weighted 0.5 × 1 × 1, respectively. S0yGN combines information from different modalities to improve the quality of gray and white matter mappings because where information may be locally homogeneous in one modality it is heterogeneous in another modality. The resulting high-resolution (1.25 mm^3^ isotropic) multimodal templates are viewable in Figure 1.

#### Reconstruction.

The 417 HARDI HCP datasets were reconstructed using generalized q-sampling imaging (GQI) with a mean diffusion distance of 1.25 mm using five fiber orientations per voxel (Yeh et al., 2010). They were also reconstructed using constrained spherical deconvolution (CSD) with a maximum harmonic order of 8 (Tournier et al., 2004). The largest b-value shell was used during reconstruction.

### Spatial Normalization of Diffusion Scans

#### Direct streamline normalization.

The remaining 400 HCP subjects T1w, T2w, and GFA volumes were spatially normalized to our template by using the same registration parameters that were used in the template creation process. The chosen SyGN parameters for both template creation and registration are a 0.1 gradient step size, cross-correlation as the similarity metric, time based SyN with symmetric gradient estimation (t = SY), with a maximum number of iterations of 100 × 100 × 50 from the coarsest to the finest level of the pyramid. Each streamline consists of a set of (*x*, *y*, *z*) coordinates in tract space. The streamline coordinates are then converted into the subject’s native voxel coordinates, allowing the application of the warps from the registration. The subject’s streamlines were estimated using ODF maxima from GQI reconstructed data. They were directly warped into the multimodal template space with a single interpolation by applying the affine and deformation field simultaneously to each (*x*, *y*, *z*) point coordinate of each streamline, preserving the native tract structure. Normalization steps were performed using Advanced Normalization Tools (ANTs). Our software (http://github.com/clintg6/DSN) interfaces with the warp fields generated by ANTs and can be applied to streamlines generated by any diffusion imaging technique.

#### Q-space diffeomorphic reconstruction.

QSDR is the generalized form of GQI that enables the reconstruction of ODFs in any template space. Its aim is to preserve fiber orientations and the number of diffusion spins under transformation. It is universal and works with DTI, single and multishell HARDI, and DSI (Yeh & Tseng, 2011). QSDR first reconstructs the raw diffusion data by using GQI and extracts the primary QA volume, which is then registered to the MNI QA template. The raw DWIs are then normalized into the MNI space by using the same transformation. The Jacobians outputted from the deformation are then used to properly reorient the b-vectors locally as each voxel’s ODF is reconstructed in the template space. The 417 HARDI HCP datasets were separately normalized into 1-mm MNI space by using QSDR with a mean diffusion distance of 1.25 mm and five fiber orientations per voxel. Attempts were made to reconstruct into our custom 1.25-mm^3^ template space, but the SPM normalization algorithms in QSDR failed to converge to our template even with heavy regularization. Resorting to the built-in method, the *R*^2^ value from the subject’s QA to the MNI template QA was on average 81, suggesting the SPM normalization performed well with the built-in MNI QA template.

#### FOD reorientation.

FODs generated from CSD in mrTrix were reoriented using apodized PSFs (Tournier et al., 2012). Specifically, each FOD is decomposed into a series of weighted spherical harmonic PSFs. The amplitude of the negative lobes of the PSFs are reduced, then each PSF is reoriented using the local affine transformation estimated from the Jacobian of the total deformation field, and finally recombined into the full reoriented FOD (Raffelt et al., 2012). The warps used for reorientation were the ANTs outputs from each subject’s symmetric diffeomorphic registration to our custom multimodal template.

#### Tractography.

Fiber tracking was performed in DSI Studio with an angular cutoff of 60°, step size of 1/2 the voxel length, minimum length of 10 mm, smoothing of 0.0, maximum length of 420 mm. FODs/reoriented FODs were converted into DSI Studio format by identifying the three largest peaks, with 60 directions for peak finding. An improved and top performing (ISMRM 2015 Tractometer Challenge) deterministic fiber tracking algorithm was used until 100,000 streamlines were reconstructed for each subject (Yeh et al., 2010).

### Network Construction

**Figure 2.**
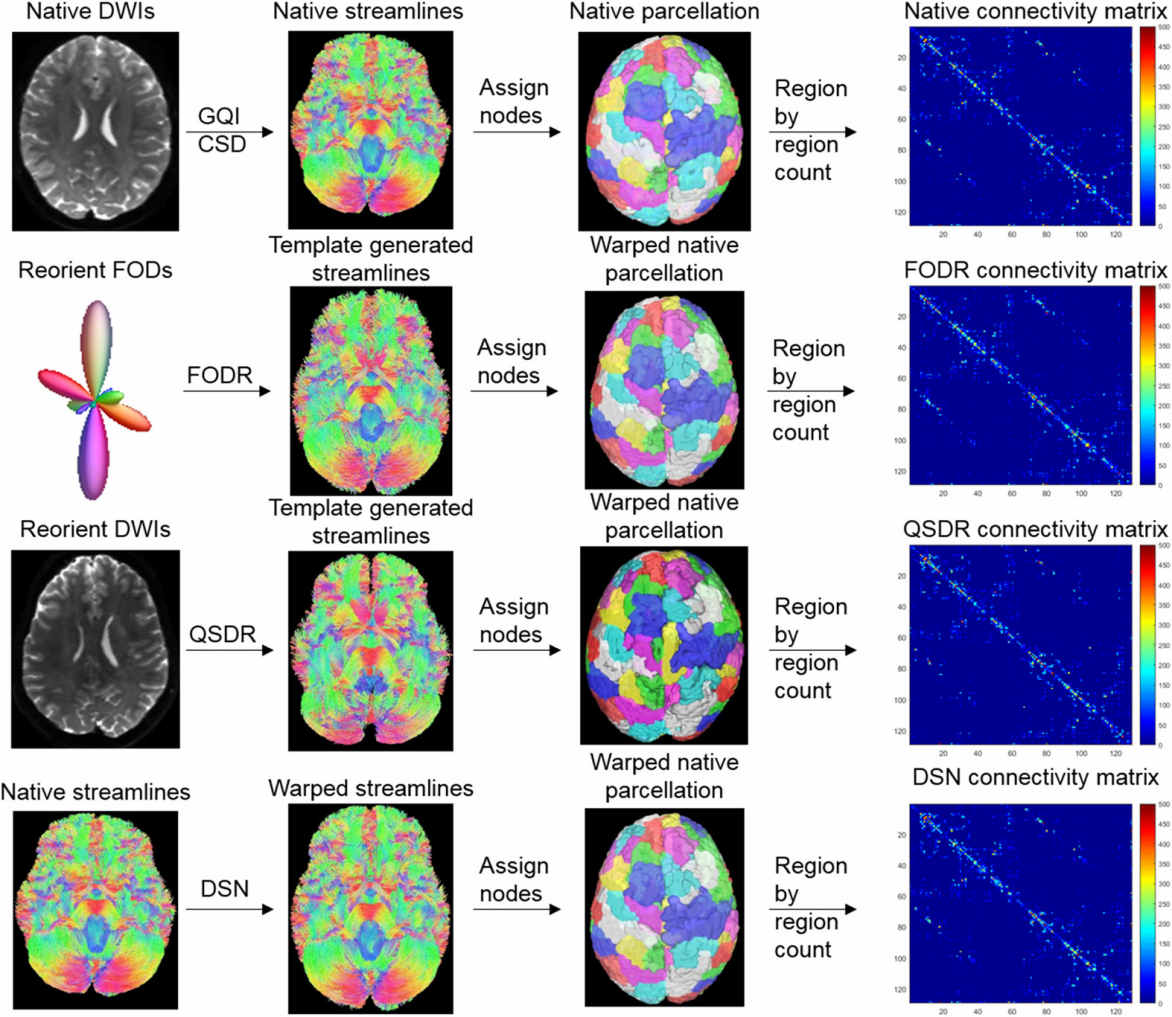
Schematic of network construction in native and template space for a single subject. Native space HCP HARDI data was reconstructed using GQI and CSD for each subject. Deterministic tractography was performed separately for the GQI and CSD reconstructed native space data. The native space data was reconstructed using CSD to fairly compare the impact of FODR. The scale 60 Lausanne parcellation was applied to assign nodes to the streamlines. The structural connectivity matrix was weighted using streamline count. For FODR, the native space FODs reconstructed using CSD were reoriented according to the deformation field output by ANTs. The subject’s native space parcellation was warped into the template space by using the same deformation field output by ANTs. The raw HARDI data was reoriented as well as the b-vectors to reconstruct ODFs in template space for QSDR. The subject’s native space parcellation was warped into the template space by using QSDR’s internal mapping. Deterministic tractography was performed for FODR and QSDR after normalization. The native space streamlines generated from ODFs reconstructed using GQI were directly warped into template space by using the deformation field output by ANTs. Node assignment and network construction for FODR, QSDR, and DSN follow the same workflow as in native space. After the connectivity matrices are constructed, the impact of different spatial normalization approaches can be measured by comparing the similarity of the connectivity matrices and network metrics derived from them for the same subject before and after normalization.

Although there are many methods for spatially normalizing data, none to our knowledge has measured the impact on a diffusion-based structural brain network. To investigate the impact on structural networks, we constructed connectivity matrices in native space by using GQI, native space using CSD, template space via DSN, template space via FOD reorientation, and MNI space via QSDR. A schematic of the workflow can be found in Figure 2.

#### Parcellation.

T1 anatomical scans were segmented using FreeSurfer (Dale et al., 1999) and parcellated according to the Lausanne 2008 atlas (Daducci et al., 2012; Hagmann et al., 2008) included in the connectome mapping toolkit. We rigidly registered the scale 60 (129 regions) parcellation to the b0 volume from each subject’s HARDI data for network construction.

We used an interpolation-free approach for directly transforming the parcellation ROIs into the template space. For every native space voxel containing a label, v_n_ = [x_n_, y_n_, z_n_], it’s coordinates are transformed directly into the template space by using the subject’s ANTs transformations as v_t_ = [x_t_, y_t_, z_t_] and the native space label is carried over to the new coordinate, v_t_. For QSDR, we used the output voxel mapping from DSI Studio to directly transform the parcellation ROIs into MNI QA template space.

#### Connectivity matrix.

To attain regional-based connectivity, a set of *N* = 129 brain region masks from the Lausanne scale 60 atlas were applied to the reconstructed fiber tracts. We determined the number of tracts that originate in one mask, *i*, and terminate in another mask, *j*, for all possible pairs of *N* masks, creating an *N* × *N* interregional anatomical connectivity matrix, M_*ij*_, where the value of any element of the matrix *M*_*ij*_ is equal to the count of tracts originating in mask *i* and terminating in mask *j*. These matrices were constructed for each of the 417 subjects’ native space GQI, native space CSD, QSDR, and FOD reoriented, and DSN streamline sets using streamline count between region pairs. The parcellations and tracking parameters were the same for all datasets and methods.

### Similarity Measures

To test the disagreement between connectivity matrices constructed in native space versus template space for the various methods, we chose the generalized Jaccard distance. This distance is a natural generalization of the Jaccard distance over sets with weighted elements. It is defined as $D_{j}=1-\frac{\sum_{i=l}^{n}\text{min}(N_{i},T_{i})}{\sum_{i=l}^{n}\text{max}(N_{i},T_{i})}$, where *N*, *T* are flattened native space and template space connectivity matrices and varies between [0, 1]. A value of 0 for Jaccard distance indicates complete similarity between native space and template space connectivity, whereas a value of 1 means complete dissimilarity. Prior work has shown this distance to satisfy the triangle inequality (Charikar, 2002).

### Network Analysis

Weighted network properties such as density and assortativity were estimated using the Brain Connectivity Toolbox (Rubinov et al., 2009). These network measures were used to further characterize the impact spatial normalization has on structural brain networks.

#### Average degree.

The average degree of a network, $\langle k\rangle =\frac{2E}{N}$, is the mean of the degree distribution and is closely related to network density. To capture the intersubject variability in degree distribution before and after normalization, we computed the difference in mean degree distribution between native and template 〈*k*_*N*_〉 − 〈*k*_*T*_〉 for each subject.

#### Density.

The network density, *D*, is defined as the number of nonzero edges in the network, *E*, divided by the total number of possible edges in the network $D=\frac{2E}{N(N-1)}$, where *N* is the number of nodes in the network, or in this case the number of or brain regions. The density is therefore proportional to the total number of connected pairs of brain regions, irrespective of the number of tracts passing between those pairs.

#### Assortativity.

The assortativity measures the preference of a brain region to connect to other brain regions of similar degree (leading to an assortative network, *A* > 0) or to other brain regions of very different degree (leading to a disassortative network, *A* < 0). The assortativity of a network is defined as,

$$A=\frac{E^{-1}\overset{n}{\underset{i=1}{\sum }}j_{i}k_{i}-{\left[E^{-1},\overset{n}{\underset{i=1}{\sum }},\frac{1}{2},\left(j_{i},{+k}_{i}\right)\right]}^{2}}{E^{-1}\overset{n}{\underset{i=1}{\sum }}\frac{1}{2}\left(j_{i}^{2},+,k_{i}^{2}\right)-{\left[E^{-1}\overset{n}{\underset{i=1}{\sum }}\frac{1}{2}\left(j_{i},{+k}_{i}\right)\right]}^{2}}$$

where *j*_*i*_, *k*_*i*_ are the degrees of the nodes at either end of the *i*th edge, with *i* = 1 … E.

Social networks are commonly found to be assortative, whereas networks such as the internet, World Wide Web, protein interaction networks, food webs, and the neural network of C. elegans are disassortative (Bassett et al., 2011).

### Effect Size

We estimated effect size by using Glass’s delta estimator that uses only the standard deviation from native space, $\Delta =\frac{\mu_{N}-\mu_{T}}{s_{N}}$, where *μ*_*N*_ is the native space mean, *μ*_*T*_ is the template space mean, and *s*_*N*_ is the standard deviation of the native space group.

### Structural Network Variability

We estimated structural network variability across 84 identical twins, 70 fraternal twins, 54 nontwin siblings, and 84 nonrelated subjects (randomly sampled from exhaustive pairing, _229_C_2_) by measuring the generalized Jaccard distance of the respective pairs’ connectivity matrices and the *R*^2^ of metrics derived from them to provide a frame of reference for the variability introduced by different spatial normalization approaches into structural networks after normalization.

## RESULTS

### Visual Comparison

**Figure 3.**
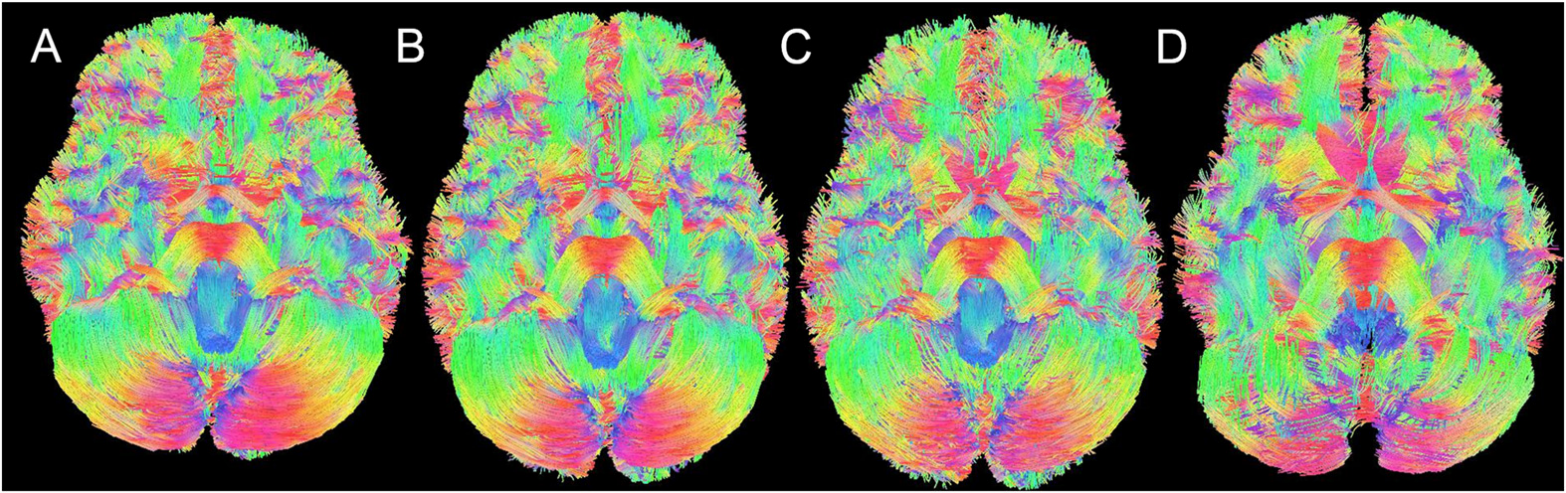
Full brain tractography from left to right for a single subject: (A) Native space, (B) DSN, (C) FOD reorientation, and (D) QSDR. Our DSN tract set most closely resembles the Native set. QSDR shows the least resemblance, followed by FOD reorientation to the native set. There is a large discrepancy in the optic chiasm region for FOD reorientation and QSDR compared with native space. DSN most closely preserves the native tract structure.

Whole brain tractograms generated using the same streamline construction parameters for a single HCP subject are visible in Figure 3 for (A) Native space, (B) DSN, (C) FOD reorientation, and (D) QSDR. From visual comparison of local or global features, it is apparent that the DSN tract set most closely resembles the Native set. The QSDR set least resembles the Native set, followed by FOD reorientation. We can see this by closely examining the region near the optic chiasm where a large discrepancy is apparent for FOD reorientation and QSDR with respect to Native space. Comparison of endpoints, where streamlines terminate at the gray-white boundary also demonstrate that DSN markedly preserves the Native branching structure compared with the other normalization methods.

**Figure 4.**
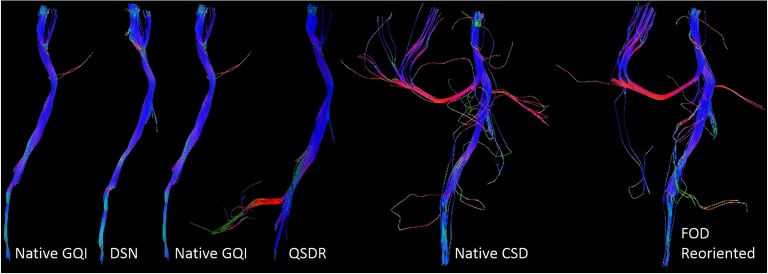
Effect of spatial normalization on the structure of the CST. Streamlines were selected using the same Native space ROI warped according to the deformation field from the normalization for each method. It is obvious from visual comparison that DSN strongly preserves the native structure compared with QSDR and FODR, which introduce variation that is not seen in their Native space counterparts. DSN most closely matches the branching in Native space compared with QSDR and FODR.

In addition to this global inspection, we also considered the impact of the normalization scheme on a predefined tract. We used a single ROI, first applied in Native space, to define the corticospinal tract (CST) in a single subject, as shown in Figure 4. The same ROI defined in Native space was warped into each respective template space by using the deformation field from the normalization, and the CST for each method was identified. DSN again excellently preserves the native tract structure in template space, visually outperforming the other normalization methods. QSDR and FODR introduce significantly more variation in the tract structure relative to native space. Notice that the branching seen in Native space is most closely matched for DSN compared with QSDR or FODR. The Native CSD set uses a different reconstruction technique compared with the Native GQI, resulting in a fundamentally different representation of the CST.

### Quantitative Comparison

To quantify the degree of variation that the different normalization methods introduce, we measured the generalized Jaccard distance between 417 subjects’ native space connectivity matrices before and after normalization. This distance measures the normalized similarity between native space connectivity and template space connectivity, such that a distance of 0 is complete similarity and 1 is complete dissimilarity.

**Figure 5.**
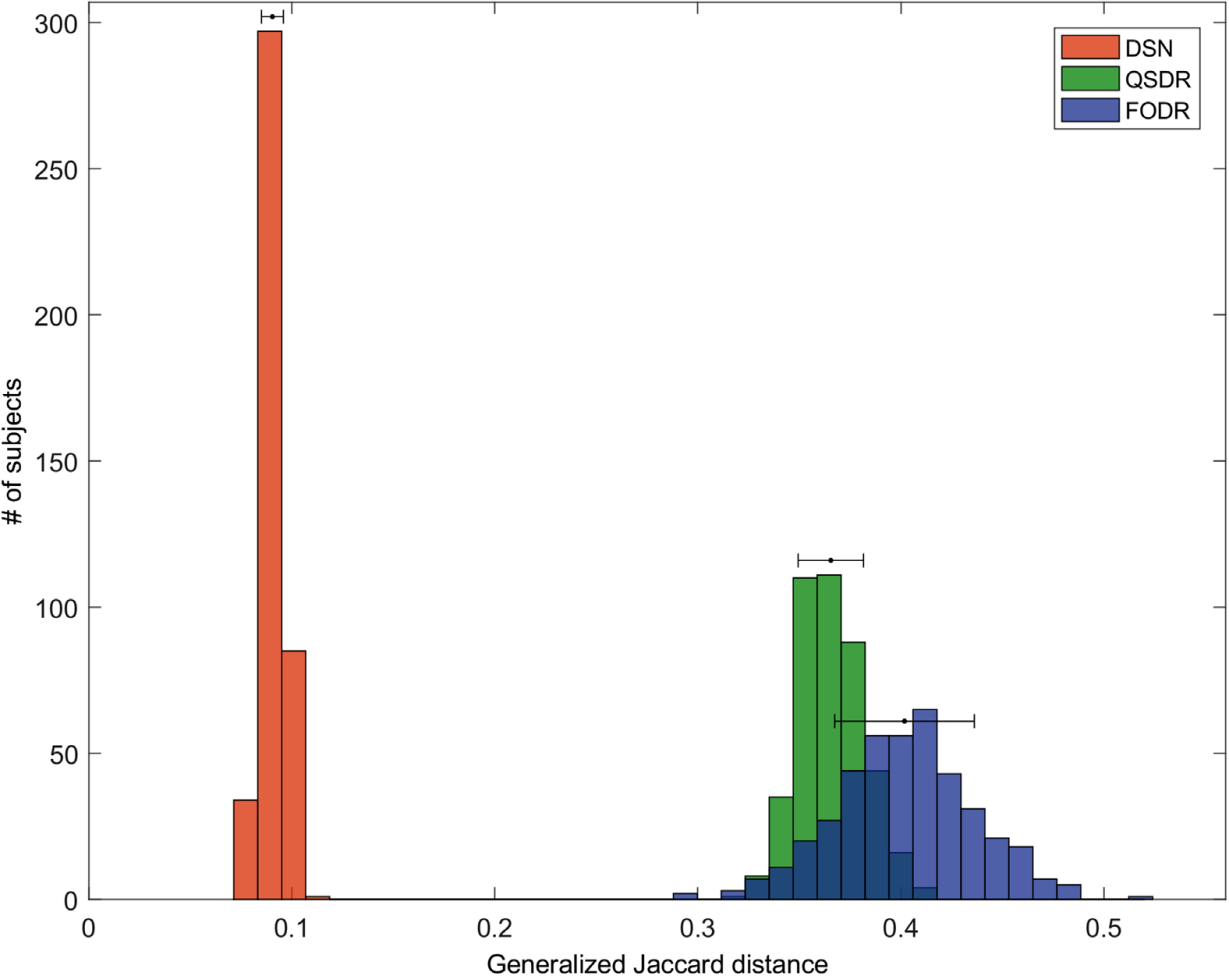
Histograms for all HCP subjects for generalized Jaccard distance between a subject’s native space connectivity matrices and template space connectivity matrices for the different spatial normalization methods. DSN (red) significantly outperforms QSDR (green) and FODR (blue). Mean + SD error bars are plotted above each distribution (black). There is no overlap between the DSN distance distribution and the QSDR and FODR distributions. The mean DSN distance is much smaller at 0.09 compared with 0.37 and 0.40 for QSDR and FODR. DSN distribution also has a narrower spread with a standard deviation of 0.005 compared with QSDR and FODR whose standard deviations are 0.018 and 0.034, respectively. Under one-way ANOVA, DSN distances test significantly smaller than QSDR and FODR with a *p*-value < 2^−16^.

Histograms of the distance for each method are plotted in Figure 5. DSN significantly outperforms QSDR and FOD reorientation in preserving connectivity (one-way repeated measures ANOVA, *p* < 2^−16^). Summary statistics are available in Table 1. The mean distance for DSN is much smaller at 0.09 compared with QSDR and FODR 0.37 and 0.40, respectively. There is no overlap between the DSN distance distribution and the other methods’ distributions. Moreover, the spread of the DSN distribution is also much tighter compared with QSDR or FODR, with a standard deviation of 0.005 versus 0.016 and 0.034. Mean + SD error bars are plotted above each distribution in Figure 5.

**Table 1.** Difference in average network degree and network metric statistics summarized for the three methods. Each measure compares the same subject’s network before and after normalization. Changes to network metrics are described by effect sizes (Δ).

**Method**	**Measurement**									
	〈*k*_*N*_〉 − 〈*k*_*T*_〉		Density				Assortativity			
	*μ*	√*σ*	*μ*	*R*^2^	*p*	Δ	*μ*	*R*^2^	*p*	Δ
DSN	−0.27	0.17	0.29	0.99	0.0006	0.24	−0.015	0.90	0.99	6.4e-5
QSDR	4.54	0.95	0.39	0.66	5.1^−289^	4.04	0.0064	0.50	2.2^−86^	3.17
FODR	14.43	3.18	0.21	0.82	1.3^−202^	2.59	−0.019	0.11	6.4^−14^	1.07

**Figure 6.**
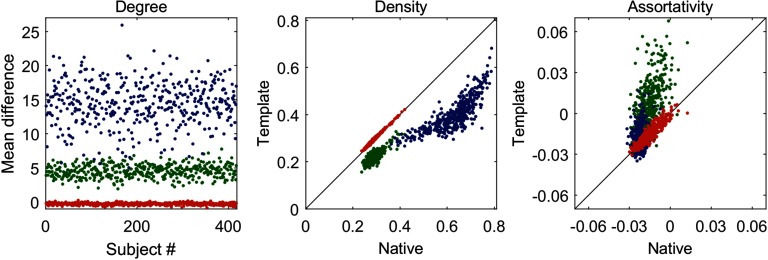
Scatter plots from left to right: difference in average network degree between native space and template space and network density and network assortativity for native space vs. template space for DSN (red), QSDR (green), and FODR (blue). Unity slope line (black). There is excellent agreement between native space networks and template space networks normalized using DSN compared with QSDR and FODR. Statistics for the metrics are summarized in Table 1.

We also measured and plotted the effect of normalization on typical structural brain network measures, such as average network degree and network density and assortativity in Figure 6 for each subject and method: DSN (red), QSDR (green), and FODR (blue); summarized in Table 1. DSN preserves the average network degree with a mean of −0.27 compared with QSDR’s 4.54 and FODR’s 14.43 difference in edges before and after normalization (Table 1). Moreover, the standard deviation in the average difference in network degree is also much smaller for DSN, 0.17, compared with QSDR and FODR, 0.95 and 3.18, respectively. The native space network density for each subject was plotted against the network density in template space. DSN preserves network density with an *R*^2^ of 0.99 versus 0.66 and 0.82 for QSDR and FODR, respectively (Table 1). Mean network density in native space is 0.29 for GQI reconstruction and 0.62 for CSD reconstruction, showing that QSDR and FODR decrease the number of edges after normalization, whereas DSN on average does not change the number of edges. This change is also reflected in the difference in average network degree scatter plot where the QSDR and FODR plots illustrate that after normalization the number of edges decreases and remains nearly unchanged for DSN. Because average network degree and network density are linearly rescaled versions of each other (see Materials and Methods) the *R*^2^, p value, and effect size of average network degree are the same as for network density and are not included in Table 1 to reduce redundancy. DSN also dramatically outperforms the other methods at preserving network assortativity. Under Welch’s paired t test with *α* = 0.0001, FODR and QSDR significantly alter network metrics with changes characterized by large to very large effect sizes (Δ), whereas DSN only has very small to small effects and does not significantly alter them. For network assortativity the mean in native space for GQI and CSD are, respectively, −0.015 and −0.022. DSN and FODR preserve the known disassortativity of structural brain networks, whereas QSDR tends to make a subject’s network after normalization more assortative. The network statistics from the 417 subjects’ data demonstrate DSN’s improvement over QSDR and FODR at preserving networks properties after normalization.

To characterize the inherent variability in structural brain networks between different subjects, we computed the mean generalized Jaccard distance for native space and each normalization method for pairs of identical twins, fraternal twins, nontwin siblings, and unrelated subjects summarized in Table 2. As expected, in native space the mean pairwise distance between identical twins is smaller (.41–.42) compared with the other groups (.46–.50). Identical twin pairs network Jaccard distance is significantly smaller than fraternal twins, nontwin siblings, and unrelated subjects’ pairs distance for all methods (one-way repeated measures ANOVA, *p* < 0.0007). No significant difference was found among distances from fraternal twin, nontwin sibling, and unrelated subject pairs. For the three normalization methods, the mean and standard deviation of the pairwise Jaccard distances for each group are nearly identical (Table 2).

**Table 2.** Familial generalized Jaccard distance for connectivity matrices for native space and each normalization method for pairs of identical twins, fraternal twins, nontwin siblings, and exhaustively for each subject to every unrelated subject.

**Method**	**Familial Generalized Jaccard Distance**							
	Identical twins		Fraternal twins		Nontwin siblings		Not related	
	*μ*	√*σ*	*μ*	√*σ*	*μ*	√*σ*	*μ*	√*σ*
Native	0.42	0.020	0.47	0.067	0.47	0.061	0.49	0.057
DSN	0.42	0.020	0.47	0.065	0.47	0.060	0.49	0.056
QSDR	0.42	0.021	0.47	0.071	0.48	0.061	0.50	0.057
FODR	0.41	0.021	0.46	0.063	0.47	0.062	0.49	0.058

To further investigate the impact of normalization method on preserving heritable features of brain connectivity, we computed the correlation of network density and assortativity between pairs of identical twins, fraternal twins, nontwin siblings, and nonrelated subjects for native space and each normalization method. The *R*^2^ between nonrelated subjects for network density and assortativity was ∼0. For the other pair types, network density shows stronger correlations than network assortativity. Network density is significantly more similar for identical twin pairs relative to unrelated subjects, but not to other pair types in native space and for DSN (one-way repeated measures ANOVA, *p* < 0.002). With QSDR no significant difference was found for any pair type. However, for FODR significant differences were found for identical twins relative to nontwin siblings and unrelated subjects, *p* < 0.002. For network assortativity, no significant difference was found across the pair types using one-way repeated measures ANOVA for native, DSN, and FODR. A significant difference was found for QSDR between nontwin siblings and unrelated subjects, *p* < 0.02. The results, summarized in Table 3, suggest that network density is more heritable than assortativity, suggesting that genetic similarity only predicts network metric similarity up to a point. Comparing the three normalization methods, each network measure obtained with DSN most closely matches the correlations seen before normalization in native space, agreeing with the significant findings in Table 1.

**Table 3.** Familial similarity of network metrics for identical twins, fraternal twins, and non-twin siblings.

**Method**	**Familial Similarity of Network Metrics**							
	Identical twins		Fraternal twins		Nontwin siblings		Not related	
	*R*^2^							
	Assortativity	Density	Assortativity	Density	Assortativity	Density	Assortativity	Density
Native	0.52	0.75	0.46	0.29	−0.04	0.28	∼0	∼0
DSN	0.49	0.76	0.53	0.31	0.07	0.33	∼0	∼0
QSDR	0.58	0.69	0.28	0.27	0.31	0.31	∼0	∼0
FODR	0.53	0.85	0.36	0.61	0.64	0.33	∼0	∼0

The results summarized in these tables show that spatial normalization affects the structural connectivity, and depending on the chosen method, the impact can be significant. DSN does not significantly alter the two tested structural brain networks properties: network density and assortativity. QSDR and FODR significantly impact a given subject’s brain network such that the distance between that same subject’s connectivity and network metrics after normalization are more comparable to a nontwin family member rather than to themselves.

## DISCUSSION

We compared three methods for spatially normalizing streamlines reconstructed from diffusion imaging data into a standardized atlas. Two of these publically available methods rely on warping of diffusion information followed by streamline reconstruction (QSDR and FODR), and the third method, DSN, directly warps the streamlines into the template space with a single interpolation. We showed that DSN readily outperforms them at preserving key native tract structure and anatomic properties of structural brain networks after spatial normalization by using 417 HCP subjects. It also has additional advantages of being generalizable to any diffusion tractography imaging method.

Current approaches for generating streamlines after spatially normalizing DWIs for population-based analyses suffer from two significant limitations. First, most of these methods depend specifically on the diffusion sampling and reconstruction method used and do not generalize easily to other methods (Alexander et al., 2001; Zhang et al., 2006; Hong et al., 2009; Raffelt et al., 2011, 2012). QSDR overcomes these limitations by using GQI to reconstruct q-space datasets acquired through either grid or shell sampling schemes in MNI space (Yeh & Tseng, 2011). But QSDR also suffers from the second and most significant limitation that also plagues FODR and tensor reorientation approaches, distortions to the maxima of the ODF. With reorientation approaches, the maxima in the native FODs no longer correspond to the maxima in the reoriented FODs (Christiaens et al., 2012). Mean angular error (MAE) for FODR between peak orientations is ∼8° (Raffelt et al., 2012). With QSDR, it is known that the maxima be fore and after normalization do not perfectly correspond—MAE is 2.27° on simulated vertical fibers and likely higher on subject data. The distortions to the normalized QSDR ODFs are responsible for the distance between native space and template space connectivity matrices because they produce small errors in the maxima of the ODFs that accumulate into large errors in the tractogram when performing deterministic tractography, that is, producing streamlines in template space that do not match the original streamlines from native space (Lazar, 2010). How much less distortion to QSDR ODFs could be gained by using an updated multimodal registration algorithm remains an open question. Our results show significant dissimilarity with large to huge effect sizes in the same subject’s network distance and metrics after normalization with QSDR and FODR. In fact, QSDR and FODR introduce so much distortion into any given subject’s streamlines that a subject’s network after normalization would more closely resemble a nontwin family member’s brain network rather than their own.

**Figure 7.**
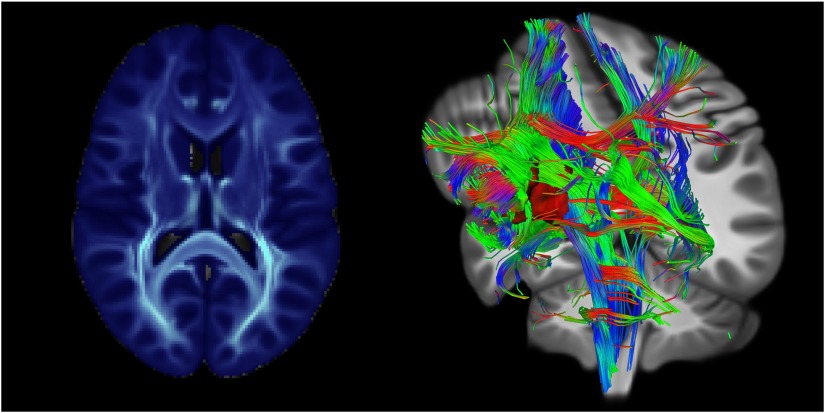
DSN supports many applications: Average EDI constructed from edge trajectories of 417 HCP subjects (left). Cortical disconnection maps can be constructed by querying streamlines that pass through a brain-injured subjects warped lesion ROI (right).

Another significant advantage of the DSN approach over QSDR is that the overall accuracy of spatial normalization can be improved by incorporating a multimodal symmetric diffeomorphic normalization framework. To do this, we first created a custom high-resolution multimodal template from 40 HCP subjects chosen through stratified random sampling to give each racial, gender, and handedness group a representation using ANTs (Avants et al., 2010). T1w, T2w, and generalized fractional anisotropy (GFA) images enhanced the fidelity and contrast of the template generation and normalization, maximizing both cortical and white matter alignment. The resulting affine and deformation field outputs from the SyN registration are applied to each (*x*, *y*, *z*) coordinate of each streamline. This reliably projects the native streamline structure into the custom template space. Moreover, because DSN accurately preserves tract structure and connectivity, it introduces important opportunities for developing a host of clinical and neuroscientific applications. Clinically, there is potential to use DSN to develop a database of anatomically consistent connectivity independent of any cortical parcellation from healthy subjects.With this, estimates of disconnected cortical regions in individual brain-injured patients can be derived as in Figure 7. Current approaches that identify network structure based solely on native space parcellations lack this ability to probe the relationship between subcortical damage and cortical disconnection at a population level. DSN enables the construction of edge density images (EDI) in Figure 7 that can be used to map network edge properties across target populations (Owen et al., 2015). Moreover, it also provides a reliable way to generate new types of cortical parcellations driven by clustering of white matter connectivity, since the tracts themselves can be spatially normalized across large populations. With this it can be used to investigate how the connectome varies across target populations, extending from the voxel scale of analysis all the way up to entire white matter pathways.

Prior work evaluating streamline normalization has highlighted the potential for adverse effects that occur with nonlinearly warping of DTI data followed by tractography (Adluru et al., 2016). Adluru and colleagues found that the least amount of overlap between native and normalized pathways occurs near the outskirts of streamlines, where the pathways begin to branch. The disparity we see in our results between structural network topology and metrics derived in native space and template space is due in part to the mismatch between branching patterns of the streamlines. The branching pattern mismatch is smallest when normalizing with DSN compared with FODR and QSDR because DSN does not rely on tracking through distorted maxima. The errors from tracking through reoriented data accumulate into the mismatch between branching patterns, which are visually obvious for CST in Figure 4. Despite their findings, Adluru et al., concluded that tracking after normalization preserves shape and produces anatomically consistent structures compared with tracking in unwarped native DTI (Adluru et al., 2016). Our results show that tracking after normalization does not reliably preserve shape, especially where streamlines begin to branch and terminate, because if it did preserve them there would be little impact to the subject’s network and its properties. It is critical that a subject’s network topology and streamline structure in native space is preserved in the template space because it is this structure that might uniquely vary across different populations and enable the characterization of differences in white matter morphology due to development, genetics, disease, or injury across populations by using tools such as LTPA (Cieslak & Grafton, 2014; Cieslak et al., 2015). Although we relied on deterministic tractography from multishell HCP HARDI data for analyzing the impact of spatial normalization on structural brain connectivity, the disruption to subject’s brain networks and streamline reconstructions are guaranteed to also apply to any sampling scheme like DTI or DSI. We chose deterministic tractography because it was applicable to all three methods and generates the same tractogram each time it is calculated, unlike probabilistic approaches, allowing us to tease apart how much of the distortion is driven by the normalization. Past work suggests that probabilistic tractography is affected less by reorientation than deterministic tractography, so if tractography is performed after normalization probabilistic methods should be employed. Nevertheless, they are also affected because of aliasing artifacts and changes to the seeding distribution that distorts the spread of the fiber population (Christiaens et al., 2012). We recommend performing tractography in native space and then normalizing with DSN to make population-based comparisons of white matter connectivity in a standardized template space.

None of the methods for spatially normalizing streamlines reconstructed from diffusion data perfectly preserve the structural brain network. The discrepancy between streamlines and networks before and after spatial normalization for FODR and QSDR are due to tracking through distorted maxima. For FODR the mismatch is due to reorientation introducing distortions to FOD maxima via lobe reshaping and interference (Christiaens et al., 2012). For QSDR, the maxima mismatch is also likely due to ODF lobe reshaping and interference. Despite DSN avoiding the issue of tracking through distorted ODFs, it is not perfect and introduces slight perturbations into subjects’ networks after normalization. These perturbations are a result of a small subset of each subjects’s native space voxels (*μ* = 1.03%) containing different parcellation labels being transformed to the same voxel coordinate in the standardized space. This is an unavoidable consequence of resampling a parcellation into a new volume because certain locations in a subject’s brain that undergo compression and expansion during the normalization can result in a subset of voxels being mapped to the same voxel coordinate in the template space. If this occurs near the interface between different parcellation labels, then a voxel in the template space that has multiple voxels mapped to it can be assigned multiple labels, where the final label at that voxel is the last label it was assigned. When the connectivity matrix is constructed in the standardized space, a small subset of the streamlines passing through these voxels can be assigned a new label that does not match the original native space label. Since the Jaccard distance and network metrics depend on the preservation of streamline count, a slight discrepancy results between native and template space DSN networks. These relabeling issues do not affect FODR and QSDR connectivity matrices because the streamlines constructed in the template space and consequently the connectivity matrices are unique relative to the native space ones.

Changes induced by QSDR and FODR to network density are also due to a subset of the streamlines not connecting pairs of nodes. Even streamlines in native space can be unassigned but the proportion increases when tracking after normalization, reducing the number of edges in the structural network. The increase in sparsity is also evident in the difference in average node degree in Figure 6, where the node degree on average is 5 edges less for QSDR and 14 edges less for FODR in the standardized space. It is unclear why QSDR and FODR reduce the density after normalization and why QSDR increases the assortativity relative to native space.

Using different relatively coarse measures of white matter connectivity, we showed that structural brain network similarity and density are strongly heritable across monozygotic twin pairs. Similarity of white matter morphology, such as obvious similarity in the shape of the corpus callosum has been described previously (Gazzaniga, 1989). Our results extend this morphologic observation by demonstrating that estimates of interregional connectivity are also driven by heritability. However, the lack of statistical significance for similarity and metrics for identical twins to other family pair types is likely due to the small sample sizes and the metrics not being heritable or too crude. Further analysis of the heritability metrics is beyond the scope of our current investigation. Future studies will be needed to determine if this heritability is a global feature or a property of specific circuits.

DSN overcomes both of the limitations of QSDR and FOD reorientation. DWIs can be acquired with any desired sampling scheme. Diffusion tensors, FODs, or ODFs can also be reconstructed using any desired method and streamlines generated using any algorithm. Most importantly, it avoids the problem of generating tracts from reoriented diffusion tensors, FODs, or ODFs that are distorted relative to their native counterparts because the spatial warping is applied directly to the streamlines. Our results show that DSN has minimal influence on basic tractography measures such as tract count and structure and does not significantly alter network metrics or topologic organization with only very small to small effect sizes. We have developed a universal framework in Python that works with most diffusion software platforms, algorithms, and ANTs for spatial normalization. It is publically available at http://github.com/clintg6/DSN (Greene, Cieslak, & Grafton, 2018).

## ACKNOWLEDGMENTS

The authors thank Allison Shapiro for the statistical analysis.

## AUTHOR CONTRIBUTIONS

Clint Greene: Conceptualization; Data curation; Formal analysis; Investigation; Methodology; Software; Visualization; Writing – original draft. Scott T Grafton: Funding acquisition; Supervision; Writing – review & editing. Matt Cieslak: Conceptualization; Data curation; Methodology; Software; Writing – review & editing.

## FUNDING INFORMATION

Scott T. Grafton, Army Research Office (http://dx.doi.org/10.13039/100000183), Award ID: W911NF-09-0001. Scott T Grafton, GE/NFL, Award ID: Head Health Challenge.

## TECHNICAL TERMS

Fiber orientation distribution (FOD): Probability distribution of fiber populations at a given orientation.
High angular resolution diffusion imaging (HARDI): Spherical shell–style sampling scheme of q-space.
Fiber orientation distribution reorientation (FODR): Normalizes fiber orientation distributions into a standardized space.
Orientation distribution function (ODF): Probability distribution of spin diffusion at a given orientation.
Diffusion spectrum imaging (DSI): Cartesian grid style sampling scheme of q-space.
Q-space: 3D space where the probability of diffusion of spins is encoded using gradients.
Q-space diffeomorphic reconstruction (QSDR): Normalizes orientation distribution functions into a standardized space.
Generalized q-sampling imaging (GQI): Method that reconstructs orientation distribution functions from spherical or cartesian shell data.
Constrained spherical deconvolution (CSD): Method that reconstructs fiber orientation distributions from spherical shell data.
Symmetric diffeomorphic normalization: An unbiased spatial normalization method that removes the effects of intersubject anatomical variability due to differences in brain size and shape.
